# Clinical and Etiological Profiles of Patients With Pancytopenia in a Tertiary Care Hospital

**DOI:** 10.7759/cureus.30449

**Published:** 2022-10-18

**Authors:** Suhas S Gajbhiye, Akash R Karwa, Avinash Dhok, Swaragandha S Jadhav

**Affiliations:** 1 Department of Internal Medicine, NKP Salve Institute of Medical Sciences and Research Center, Nagpur, IND; 2 Department of Radiodiagnosis, NKP Salve Institute of Medical Sciences and Research Center, Nagpur, IND

**Keywords:** megaloblastic anemia, fever, fatigue, pallor, aplastic anemia, pancytopenia

## Abstract

Background

Pancytopenia is more of a manifestation of a spectrum of underlying diseases affecting the bone marrow. Specific treatment relies solely on early diagnosis and identification of the accurate etiology. We aimed to generate data on the clinical and etiological profiles of patients diagnosed with pancytopenia.

Materials and methods

Fifty patients more than 13 years of age with pancytopenia who reported to a tertiary care hospital were included in the study. Thorough clinical examination, hematological investigation, and bone marrow biopsies were performed, and relevant data were recorded and analyzed statistically.

Results

Pancytopenia was most common in the age group of 25-34 years, with a male preponderance. The most common presenting complaints were fatigue and fever, with pallor present in all patients, followed by splenomegaly and hepatomegaly in a few patients. Aplastic anemia is the most common cause of pancytopenia, followed by megaloblastic anemia and leukemia.

Conclusion

While fatigue and fever are the most usual symptoms of pancytopenia, clinical pallor, hepatomegaly, and splenomegaly may be evident. Among the several etiologies, aplastic anemia is one of the most common causes of pancytopenia.

## Introduction

Pancytopenia, a clinical-hematological entity, is a bone marrow disorder that is frequently encountered in clinical practice. It is more of a clinical manifestation due to a spectrum of diseases affecting the bone marrow and/or the white blood cells (WBCs), red blood cells (RBCs), as well as platelets, rather than just a disease entity [1]. Depending upon the severity of anemia, leukopenia, and thrombocytopenia, its clinical presentations vary. Generalized weakness, fever, weight loss, abnormal bleeding tendencies, shortness of breath, etc. are the usual manifestations of pancytopenia, and the prognosis depends on the correct and timely diagnosis of the underlying etiology [2].

Regarding the clinical manifestations of pancytopenia, it is a manifestation of various prime malignant as well as non-malignant clinical disorders. Decreased production of hematopoietic cells, such as in aplastic anemia, abnormal cells infiltrating the bone marrow, such as in hematological malignancies, autoimmune disorders, hypersplenism, excess cell destruction due to ineffective production such as in megaloblastic anemia, etc. are few of the many possible mechanisms behind the development of pancytopenia [3].

The majority of the cases of pancytopenia are cured with the specific treatment guided by the cause as well as the extent of severity of the disease or, at times, the cases would require the timely initiation of supportive treatment to reduce morbidity and mortality, thereby improving the quality of life [4].

Since the cause and clinical presentations largely dictate the treatment plan and prognosis of patients with pancytopenia, we aimed to generate a thorough clinical and etiological profile of patients with pancytopenia reporting to a tertiary care hospital.

## Materials and methods

This cross-sectional study was conducted over a period of one year from August 2014 to July 2015 at the departments of General Medicine and General Pathology, Calcutta National Medical College and Hospital, Kolkata, on 50 patients above 13 years of age selected by the convenience sampling method, admitted to the hospital, with a hemoglobin concentration of <10 mg%, absolute neutrophilic count <1,800/mm^3^, and platelets <100,000/mm^3^. The institutional ethical committee, Calcutta National Medical College, Kolkata, 01/03/01/14, approved the study, and we also acquired written informed consent from all patients included in the study. None of the patients refused to consent to participate in our study.

A detailed clinical history was documented, followed by a comprehensive assessment of the patients through physical examination and laboratory investigations. The patients were advised mandatory investigations, including complete blood count, peripheral blood smear, erythrocyte sedimentation rate (ESR), liver function tests (LFT), urine routine microscopy, bone marrow aspiration study, bone radiographs, chest radiographs, ultrasonography, and tests for venereal diseases. They were further advised to analyze vitamin B12, folic acid, free T4 (FT4), and thyroid stimulating hormone (TSH) levels.

Based on the clinical and biochemical analyses, the etiology of pancytopenia in each patient was profiled.

Statistical analysis

The data were tabulated in Microsoft Office Excel (Microsoft Corporation, Redmond, WA) and analyzed using GraphPad Instat 3 software (GraphPad Software, San Diego, CA). The chi-square test was used to analyze the association between two categorical variables, and a p-value of <0.05 was statistically significant.

## Results

The age of the 50 patients diagnosed with pancytopenia ranged from 13 to 75 years, with the maximum number of patients in the age group of 25-34 years, followed by the group of 55-64 years, with a mean age of presentation of 42.3 years (Table 1).

**Table 1:** Age distribution of the study patients

Age Group (in years)	Patients	
	N	%
13-24	09	18
25-34	12	24
35-44	05	10
45-54	09	18
55-64	10	20
≥65	05	10

The majority of patients were males (56%), with a male-to-female ratio of 1.27 (Table 2). 

**Table 2:** Sex distribution of the study patients

Sex	Patients	
	N	%
Male	28	56
Female	22	44

Furthermore, the majority of the patients (60%) belonged to rural areas (Table 3).

**Table 3:** Urban and rural distribution of the study patients

	Male	Female	Total	%
Urban	12	08	20	40
Rural	16	14	30	60
	28	22	50	100

The most frequent clinical manifestation in the patients included in the study was fatigue (78%), followed by fever, petechiae/purpura, bleeding gums, hematemesis, and melena (Table 4).

**Table 4:** Distribution of symptoms among the study patients

Symptoms	N	%
Fatigue	39	78
Fever	34	68
Bleeding Gum	7	14
Petechia/purpura	12	24
Hematemesis	5	10
Malena	4	8

On physical examination, all patients with pancytopenia included in our study exhibited pallor, with almost half of them exhibiting splenomegaly; a few exhibiting hepatomegaly, sternal tenderness, pigmentation, and lymphadenopathy; and the other few exhibiting glossitis, icterus, and ascites (Table 5).

**Table 5:** Physical findings in the study patients

Physical findings	N	%
Pallor	50	100
Pigmentation	7	14
Icterus	5	10
Lymphadenopathy	7	14
Ascites	4	8
Hepatomegaly	12	24
Splenomegaly	20	40
Sternal Tenderness	11	22
Glossitis	9	18

The absolute neutrophil count in the patients ranged from 168 to 1,750 cells/mm^3^, with the majority having a count between 501 and 1,000 cells/mm^3^, with a maximum of them having a fever; however, no statistically significant relationship existed between the absolute neutrophil count and the incidence of fever (Table 6).

**Table 6:** Correlation between absolute neutrophil count and fever in the study patients

ANC	Patients with fever	Patients without fever	N	%
0-500	6	3	9	18
501-1000	14	2	16	32
1001-1500	7	4	11	22
>1500	7	7	14	28
Total	34	16	50	100

The hemoglobin levels in the study patients ranged from 2.3 gm% to 9.2 gm%, with a mean concentration of 6.35%. The majority of patients (56%) had moderate anemia, followed by others exhibiting severe (24%) or mild (20%) anemia (Table 7).

**Table 7:** Hemoglobin concentration in the study patients

Hb gm%	Severity of anemia	N	%
≤6	Severe	12	24
6.1 to 8	Moderate	28	56
8.1 to 10	Mild	10	20

The total leukocyte count ranged from 500-3,800 cells/mm^3^, with a mean of 2,160 cells/mm^3^. The majority of the patients (76%) had a moderate degree of leukopenia, followed by 14% of the patients having severe leukopenia and 10% having mild leukopenia. Severe leukopenia was more common in the age group of 45-65 years (Table 8).

**Table 8:** Total leukocyte count distribution in the study patients and correlation with the age of the patients

Age group	Mild (>3000 cells/mm^3^)	Moderate (1000-3000 cells/mm^3^)	Severe (<1000 cells/mm^3^)
13-24	02(4%)	06(12%)	1
25-34	00	12(24%)	0
35-44	00	04(8%)	1
45-54	01(2%)	06(12%)	2
55-64	01(2%)	07(14%)	2
≥65	01(2%)	03 (6%)	1
Total	05(10%)	38(76%)	7

The platelet count ranged from 4,000-900,000 cells/mm^3^, with the majority of the patients (42%) having a platelet count ≤25,000 cells/mm^3^ and statistically significant more bleeding tendencies as compared to the patients with platelets above this count (Table 9). 

**Table 9:** Platelet count and the correlation with bleeding tendencies in the study patients

Platelet count	Patients with bleeding manifestations	Patients without bleeding manifestations	N	%
≤25000	14	7	21	42
26000-5 0000	04	05	09	18
51000-7 5000	03	9	12	24
76000-1 00000	00	08	08	16
Total	21	29	50	100

A majority of the patients (46%) had hypocellular bone marrow, followed by 38% with hypercellular bone marrow and 16% with normocellular bone marrow. Thus, it could be inferred that aplastic anemia was the common cause of pancytopenia in most patients (Table 10).

**Table 10:** Bone marrow cellularity in the study patients

Bone Marrow cellularity	N	%
Hypocellular	23	46
Hypercellular	19	38
Normocellular	08	16

Among all the etiological factors for pancytopenia, aplastic anemia was the most common (36%), followed by megaloblastic anemia, acute leukemia, myelodysplastic syndrome, and chronic liver disease (Table 11).

**Table 11:** Etiology for pancytopenia in the study patients

Etiology	N	%
Aplastic Amenia	18	36
Megaloblastic Anemia	11	22
Acute Leukemia	4	8
Myelodysplastic Syndrome	3	6
Chronic Liver Disease	3	6
Multiple Myeloma	2	4
Malaria	2	4
Typhoid Fever	2	4
Dengue Fever	2	4
Systemic Lupus Erythematosus (SLE)	2	4
Hypersplenism	1	2

## Discussion

Red blood cells, white blood cells, and platelets, the three cellular components of peripheral blood, are reduced in pancytopenia. Pancytopenia itself is a manifestation of several other clinical disease entities; successful treatment relies on early identification of the condition through clinical presentation and accurate recognition of the etiological parameter. To broaden this knowledge, we studied the clinical and etiological profiles of 50 patients with pancytopenia.

The male-to-female ratio among patients with pancytopenia in our study was 1.27:1, which was concurrent with the findings of Hagler et al., who proposed a male-to-female ratio of 1.2:1 [5]. Likewise, the majority of the patients in our study were between the ages of 25 and 34 years. In the study conducted by Hagler et al., the patients belonged mainly to the age group of 21-40 years [5]. In addition, the studies conducted by Khodke K et al. and Khunger et al. proposed male-to-female ratios of 1.3:1 and 1.2:1, respectively [6,7].

In our study, most of the patients belonged to the rural areas of West Bengal. In our study, most of the patients belonged to the rural areas of West Bengal. This could be attributable to nutritional deficiencies, especially vitamin B12 deficiencies, attributable to the low socioeconomic strata in the rural areas.

The most frequent presenting symptom was fatigue, followed by fever and bleeding tendencies. This was congruent with another study carried out by Kondal RS and Saikrishna K on 30 patients who exhibited fatigue (86%) as the most frequent presenting symptom of pancytopenia, followed by fever (70%) [8]. However, in another study conducted by Khodke K et al. on 50 patients with pancytopenia, fever (40%) was the most common presenting symptom, followed by bleeding tendencies (20%) [6]. Similarly, another study by Ramzan M et al. showed that in individuals with pancytopenia, fever (86.7%) was the most frequent presenting symptom, followed by irritability (76%) and dizziness (64%) [9]. However, we can deduce that anemia or thrombocytopenia is typically responsible for the reported symptoms.

Among the clinical signs, pallor was ubiquitously present in all patients with pancytopenia; 40% exhibited splenomegaly, 24% exhibited hepatomegaly, 22% exhibited sternal tenderness, and 14% exhibited lymphadenopathy. Furthermore, due to megaloblastic anemia, knuckle pigmentation was evident in 14% of patients. Patients with underlying chronic liver disease have icterus due to hepatocellular injury. Nutritional deficiencies result in stomatitis and glossitis (18%) in a few patients with pancytopenia. This was consistent with a study conducted by Khodke K et al. in an Indian population with pancytopenia, where pallor was observed in all patients, followed by splenomegaly (40%), hepatomegaly (38%), purpuric spots (28%), and lymphadenopathy (12%) [6]. Another study by Hamid GA and Safa AR in patients with pancytopenia in Yemen found pallor in all patients, followed by splenomegaly (44%), purpuric spots (38.6%), hepatomegaly (21.3%), and lymphadenopathy (14.6%) [10].

We found that the majority of the patients with pancytopenia (56%) had moderate anemia, followed by severe and mild anemia. Scott et al. also reported moderate anemia in the maximum number of patients in their study sample; however, the incidence of severe anemia was minimal [11]. Similar to anemia, a moderate degree of leukopenia was evident in the majority (76%) of our study patients, followed by severe and mild leukopenia. Scott et al. also reported moderate leukopenia in 64% of patients [11], and Wong KF et al. reported an even higher percentage (83.3%) of patients exhibiting moderate leukopenia [12].

We did not find any statistically significant correlation between the total number of neutrophils and the prevalence of fever in pancytopenia patients. However, Kondal RS and Saikrishna K suggested a higher risk of developing fever due to infection when the absolute neutrophil count was <500 cells/µL [8].

The majority of patients with pancytopenia (42%) had a platelet count of ≤25,000 cells/mm^3^, with >50% of the patients exhibiting bleeding tendencies and a statistically positive correlation between platelet count and bleeding tendencies. Scott et al. also reported severe thrombocytopenia in 43.6% of the patients with pancytopenia [11]. However, Wong KF et al. reported mild thrombocytopenia in most patients (83.3%), followed by moderate thrombocytopenia, and found no incidence of severe thrombocytopenia in patients with pancytopenia [12].

Bone marrow examination is an essential diagnostic technique in patients with pancytopenia. In our study, the majority of the patients (46%) had hypocellular bone marrow, followed by 38% of the patients exhibiting hypercellular bone marrow. This was quite similar to the findings of Bhandari and Satyanarayan, who reported 50% of the patients with pancytopenia, and Feng et al. who reported 39% of the patients to be exhibiting hypocellular bone marrow [13,14]. However, Al-Eissa et al. reported that 87.5% of patients with pancytopenia have hypercellular bone marrow [15]. In our study, all the cases of aplastic anemia (34%), confirmed by bone trephine biopsy, had hypoplastic/aplastic marrow, with an idiopathic etiology. Most patients with megaloblastic anemia had hypercellular bone marrow. All patients with leukemia (8%) had hypercellular bone marrow, with visible blast cells. After a detailed investigation, two of the patients turned out to have acute lymphoblastic leukemia stage L2 and two cases to have acute myelocytic leukemia. Two patients with refractory anemia turned out to have myelodysplastic syndrome on bone trephine biopsy. The bone marrow of these patients was normal to hypercellular with an increased number of immature myeloid precursor cells and the presence of ring sideroblasts with dyserythropoietic changes. Jha A et al. through their study with bone marrow examination of the patients with pancytopenia revealed hypoplastic bone marrow (29%), megaloblastic marrow (23.6%), hematological malignancies (21.6%), erythroid hyperplasia (19.6%), and normal bone marrow (6%) [16].

In our study, we found two cases of multiple myeloma with hypercellular marrow (>20% plasma cells and plasmablasts with an increased nuclear to cytoplasmic ratio), two cases of systemic lupus erythematosus with one having hypocellular and the other having normocellular bone marrow.

Varma N and Dash S in their study on patients with pancytopenia from India reported aplastic anemia to be the commonest cause (29.5%) while Jalaee KH and Keihani M reported the incidence to be 52.7% [17,18]. Acute leukemia was the cause of pancytopenia in 8% of the patients in our study and stands to be the third most common etiology, with its incidence being reported as 13.3% in a study conducted by Hamid GA and Safa AR [10]. The other etiologies of pancytopenia in our study were dengue fever and malarial fever, which responded well to the therapies with the restoration of the cytopenia. Avasthi R et al. reported one out of the 22 patients with pancytopenia in their study to have Plasmodium (P.) vivax and P. falciparum malaria [19] while Bhandari M and Satyanarayan S reported six patients to have falciparum parasitemia [13]. Alcoholic chronic liver disease and myelodysplastic syndrome were the other etiologies as well. Jalaee K et al., in their study on the Iranian population deciphered myelodysplastic syndromes as the fourth most common cause of pancytopenia, after acute leukemia, aplastic anemia, and megaloblastic anemia [18].

While we found alcoholic liver diseases to be causative of pancytopenia in two of the 50 patients, Jain A and Naniwadekar M found alcoholic liver cirrhosis in 27 of the 250 patients studied [20].

Thus, it is evident through our study and the existing literature that there are vivid etiologies behind pancytopenia, with aplastic anemia being the most evident. Our study had certain limitations, namely, a small sample size, hospital-based study design, and the fact that the majority of the study patients belonged to rural areas with low incomes, thus not being a mere representation of the society.

## Conclusions

Out of various etiologies, megaloblastic anemia and aplastic anemia are the most common etiologies. To determine etiologies, thorough clinical and hematological evaluation becomes essential, and bone marrow aspiration and biopsy become an important part of the investigation. By diagnosing pancytopenia and its etiology from the clinical presentations and investigation, management can be initiated early and the reversible causes can be treated effectively further reducing morbidity and mortality.
